# The “healthy dyad” bias in veteran-caregiver dyadic research: a methodological caution

**DOI:** 10.1093/geroni/igag028

**Published:** 2026-03-30

**Authors:** Alisa Bedrov, Erin D Bouldin, Roxana E Delgado, Mary Jo Pugh

**Affiliations:** Informatics, Decision-Enhancement and Analytic Sciences Center, VA Salt Lake City Health Care System, Salt Lake City, Utah, United States; Division of Epidemiology, Department of Internal Medicine, University of Utah Spencer Fox Eccles School of Medicine, Salt Lake City, Utah, United States; Informatics, Decision-Enhancement and Analytic Sciences Center, VA Salt Lake City Health Care System, Salt Lake City, Utah, United States; Division of Epidemiology, Department of Internal Medicine, University of Utah Spencer Fox Eccles School of Medicine, Salt Lake City, Utah, United States; School of Nursing, University of Texas San Antonio, San Antonio, Texas, United States; Informatics, Decision-Enhancement and Analytic Sciences Center, VA Salt Lake City Health Care System, Salt Lake City, Utah, United States; Division of Epidemiology, Department of Internal Medicine, University of Utah Spencer Fox Eccles School of Medicine, Salt Lake City, Utah, United States

**Keywords:** Caregiving, Veterans, Relationship data, Representative samples, Non-response bias

Innovation and Translational Significance: Caregivers are critical to supporting individuals with complex medical conditions and disabilities, bringing caregiving to the forefront of research, policy, and health initiatives. Incorporating perspectives of both caregivers and the patients they support through dyadic research provides a more comprehensive view of the interdependent dynamics within relationships. This study highlights a potential “healthy dyad” bias that systematically underrepresents caregivers who are experiencing higher caregiver burden and lower mental health and social well-being. Future studies should critically consider how caregiver-care recipient dyads are recruited and the generalizability of those findings to caregivers, patients, and other populations.

## Background and objectives

With the increasing prominence of caregiver roles in healthcare in the United States,^1^ more research studies have begun recruiting caregiver-patient *dyads* to examine how each dyad member mutually influences the other’s health and well-being.^2^ However, recruiting dyads to participate in research is more challenging than individual recruitment.^3^ Moreover, although research on caregivers has been rapidly expanding, dyadic research on veterans with complex health conditions and their caregivers remains underrepresented. Accordingly, it is important to understand the representativeness of dyadic data and consider ways to improve dyadic recruitment strategies across unique populations like military/veteran families.

The validity of dyadic research depends on complete data, yet non-response from one partner is a common challenge that can introduce systematic bias. Prior research indicates this non-response is not random; it is more likely with indirect recruitment^4^^,^^5^ and often associated with specific caregiver characteristics,^6^ couple dynamics,^7^ and the health and demographics of military service members.^8^ Accordingly, the current body of dyadic caregiver research may be based on samples that are not fully representative. To better understand this bias, our study compared key demographic, health, and relationship characteristics in veteran-caregiver dyads with complete versus incomplete data. We aimed to highlight potential limitations of generalizing from dyadic data to the entire sampled population and to consider which participant characteristics can inform more intentional dyadic recruitment efforts.

## Research design and methods

### Participants and procedure

This study is a secondary analysis using data collected as part of a larger study on the health and quality of life of veterans with and without post-traumatic epilepsy (PTE) and their caregivers.^9^ The study recruited veteran-caregiver dyads where caregivers were at least 18 years old. Using strategies like social media campaigns and recontacting previous study participants, veterans and caregivers were recruited to complete a self-report survey on health and well-being, with targeted modules on deployment history (veterans) and caregiving duties (caregivers). Dyad linking was facilitated by asking participants to refer their partner for an invitation upon survey completion.

### Outcome measures

Primary outcomes were grouped into four domains: demographics, health status, relationship functioning, and caregiving aspects. Health status included physical and mental health components from the Veteran’s RAND 12-item Health Survey (eg, “During the past 4 weeks, how much of the time has your physical health or emotional problems interfered with your social activities”). Relationship functioning included the Dyadic Relationship Scale (two subscales: positive dyadic interactions and dyadic strain);^10^ loss of self (since leaving the military/becoming a caregiver); and perceived social support. Caregiving aspects included the 12-item Zarit Caregiver Burden Scale (eg, “Do you feel strained when you are around your care recipient”) and positive aspects of caregiving. All composite measures showed good reliability. For details regarding specific measures, see Bouldin and colleagues.^9^

### Data analysis

To examine differences between (a) veterans with complete vs. incomplete dyadic data and (b) caregivers with complete vs. incomplete dyadic data, we conducted Welch’s *t*-tests for unequal sample sizes for continuous variables and chi-square tests for categorical variables. We applied familywise Bonferroni corrections based on conceptual variable groupings: for demographic and health variables, α = 0.01; for relationship and caregiving variables, α = 0.008. Cohen’s *d* and Cramér’s V were calculated as effect size estimates.

## Results

The final sample included 202 veterans and 180 caregivers, yielding 100 complete veteran-caregiver dyads (49.5% of veterans; 44.4% of caregivers). Most caregivers were the veteran’s spouse/partner. Table 1 shows results comparing veterans and caregivers from complete versus incomplete dyads. Veterans did not differ in demographics, health status, or relationship quality by whether their caregiver responded to the survey. However, veterans with a non-responding caregiver (i.e., incomplete dyadic data) reported significantly lower social support than those whose caregiver responded.

**Table 1:** Comparing veterans and caregivers in complete vs. incomplete dyads with missing partner data across key variables.

Variables	Veterans			Caregivers		
	Dyads	Non-dyads	Effect size [95% CI]	Dyads	Non-dyads	Effect size [95% CI]
***Demographics***						
**Age, *M* (*SD*)**	47.60 (11.20)	47.47 (9.35)	0.01 [–0.26, 0.29]	46.75 (11.40)	45.67 (10.25)	0.10 [–0.19, 0.39]
**Sex, *n* (%)**			0.15 [0.00, 1.00]			0.19 [0.04, 1.00]
Male	86 (86.0)	75 (72.8)		14 (14.1)	2 (2.4)	
Female	14 (14.0)	28 (27.2)		85 (85.9)	81 (97.6)	
**Race/ethnicity, *n* (%)**			0.00 [0.00, 1.00]			0.10 [0.00, 1.00]
White	74 (74.0)	72 (69.9)		80 (80.0)	70 (83.3)	
Black/African American	11 (11.0)	10 (9.7)		7 (7.0)	2 (2.4)	
Multi-racial	10 (10.0)	9 (8.7)		12 (12.0)	8 (9.5)	
Other	5 (5.0)	12 (11.7)		1 (1.0)	4 (4.8)	
**Education, *n* (%)**			0.05 [0.00, 1.00]			0.17 [0.00, 1.00]
High School/GED	19 (19.0)	12 (11.8)		17 (17.5)	4 (4.8)	
Some College	34 (34.0)	36 (35.3)		30 (30.9)	34 (41.0)	
College Graduate	26 (26.0)	36 (35.3)		32 (33.0)	26 (31.3)	
Graduate School	21 (21.0)	18 (17.6)		18 (18.6)	19 (22.9)	
**Access to health care, *n* (%)**			0.00 [0.00, 1.00]			0.00 [0.00, 1.00]
Yes	86 (86.9)	86 (85.1)		76 (76.8)	59 (71.1)	
No (cost-prohibited)	13 (13.1)	15 (13.9)		23 (23.2)	24 (28.9)	
***Health variables***						
**PCS-12**	32.74 (11.18)	32.12 (11.45)	0.05 [–0.22, 0.33]	45.15 (11.68)	44.11 (11.20)	0.09 [–0.20, 0.38]
**MCS-12**	37.42 (13.27)	36.24 (11.49)	0.10 [–0.18, 0.37]	46.11 (12.75)	38.78 (13.89)	0.55** [0.25, 0.85]
**PTSD severity**	13.50 (4.70)	12.93 (5.17)	0.11 [–0.16, 0.39]	—	—	—
**TBI status, *n* (%)**			0.04 [0.00, 1.00]			0.03 [0.00, 1.00]
Yes	70 (70.0)	64 (62)		31 (31.0)	20 (24.0)	
No	30 (30.0)	39 (38)		69 (69.0)	64 (76.0)	
**Epilepsy status, *n* (%)**			0.07 [0.00, 1.00]	—	—	—
Yes	39 (40.0)	50 (49.5)				
No	59 (60.0)	51 (50.5)				
***Relationship variables***						
**Dyadic strain**	2.25 (0.66)	2.43 (0.67)	–0.28 [–0.57, 0.00]	2.29 (0.69)	2.66 (0.65)	–0.55** [–0.85, –0.25]
**Positive dyad interactions**	2.91 (0.54)	2.88 (0.51)	0.07 [–0.21, 0.35]	2.97 (0.55)	2.73 (0.58)	0.43* [0.13, 0.72]
**Resilience**	10.57 (3.84)	10.31 (4.19)	0.06 [–0.21, 0.34]	12.72 (3.50)	12.13 (3.38)	0.17 [–0.12, 0.46]
**Perceived social support**	18.78 (5.29)	16.62 (5.88)	0.39* [0.11, 0.66]	16.27 (5.42)	13.32 (5.12)	0.56** [0.26, 0.85]
**Loss of self**	2.95 (0.90)	2.88 (0.90)	0.08 [–0.20, 0.36]	2.11 (0.93)	2.75 (0.94)	–0.69** [–0.98, –0.38]
**Thriving**	32.00 (8.12)	30.43 (7.87)	0.20 [–0.08, 0.47]	—	—	—
***Caregiving variables***						
**Caregiver burden *(continuous scale)***	—	—	—	15.59 (9.91)	23.63 (11.15)	–0.77** [–1.07, –0.46]
**Caregiver burden * (categorical bins; n (%))***	—	—	—			0.28** [0.15, 1.00]
High (scores >16)				45 (45.0)	62 (73.8)	
Low (scores ≤16)				55 (55.0)	22 (26.2)	
**Positive caregiving aspects**	—	—	—	10.05 (2.05)	9.56 (2.29)	0.23 [–0.06, 0.52]
**ADLs, *n* (%)**	—	—	—			0.00 [0.00, 1.00]
Yes				47 (47.0)	44 (52.4)	
No				53 (53.0)	40 (47.6)	
**Education interrupted due to caregiving, *n* (%)**	—	—	—			0.35** [0.22, 1.00]
Yes				16 (16.3)	41 (49.4)	
No				82 (83.7)	42 (50.6)	

Abbreviations: ADLs, activities of daily living; MCS-12, mental health component score; PCS-12, physical health component score; PTSD, post-traumatic stress disorder; TBI, traumatic brain injury. *t*-tests were used to compare continuous variables with means and standard deviations; corresponding effect size estimates and 95% confidence intervals represent Cohen’s *d*. Chi-square tests were used to compare categorical variables with frequencies; corresponding effect size estimates and 95% confidence intervals represent Cramér’s V. Due to the large number of comparisons, alpha levels were adjusted to α = 0.01 for demographic and health variables, and α = 0.008 for relationship and caregiving variables. All caregiver results held after controlling for the length of caregiving.

*p < .01. **p < .001.

Caregivers also did not differ in demographics or physical health by whether the veteran responded to the survey. However, caregivers with a non-responding veteran reported significantly worse mental health, greater loss of self, higher caregiver burden, and higher rates of educational interruption due to caregiving than those whose veteran responded. These caregivers also reported significantly worse relationship functioning and lower social support.

## Discussion and implications

These data suggest that the caregiving dyads represented in dyadic research may meaningfully differ in mental health status, relationship quality, and caregiving burden relative to dyads with missing partner data. Caregivers with higher personal and relational strain were more likely to have incomplete dyadic data, preventing insight into how the unique veteran-caregiver relationship might be associated with or affected by that strain. These findings suggest a potential “healthy dyad” bias in research that overlooks individuals and families with fewer social and emotional resources, leaving them systematically underrepresented. Other studies on couples-based interventions and health behavior similarly found that complete dyads were more likely to have better health and relationship functioning than incomplete dyads.^8^ Thus, our understanding of caregiving relationships and subsequent interventions to address health and relationship functioning may be excluding an entire subset of individuals and relationships who would stand to benefit most or whose responses to interventions might systematically differ from dyads included in research.

Future research should work to understand and address the reasons behind non-response bias. For instance, caregivers with a higher burden may need multiple modes of participating in research and potentially larger monetary or alternative incentives. Dyadic research should be flexible and intentional in survey design and administration to allow individuals with complex medical conditions to actively participate. Alternatively, caregivers with worse relationship functioning may be unwilling or hesitant to encourage their care recipient to participate. We also observed a trend toward more male caregivers among complete dyads, suggesting potential discrepancies between caregiver self-identification and patients' perceptions of male caregivers. However, given that these data were from a single sample, future research should examine whether similar biases would replicate across other settings and dyadic samples, along with contextual factors that may impede dyadic recruitment. Understanding the reasons for incomplete data is critical to the generalizability of dyadic research, as these studies may otherwise fail to capture important dyadic processes among individuals and families most at risk of poor health and relationship outcomes.

Altogether, this research underscores the importance of considering participant recruitment in populations underrepresented in dyadic research (i.e., caregivers and veterans with complex medical conditions). Existing dyadic caregiver data may be skewed toward caregivers with lower caregiver burden, generally positive relationships with their veteran, and broader social networks. More dyadic research is needed on caregiver-patient dyads and military families to inform health initiatives that can reduce caregiver burden and associated strain for both caregivers and care recipients. In tandem, we need to develop strategies to recruit and retain caregivers with higher levels of strain and fewer social resources.

## Funding

This work was supported by the Department of Defense Congressionally Directed Medical Research Program: Epilepsy Research Program (W81XWH-20-1-0764); the VA Health Systems Research, Research Career Scientist award (1IK6HX003762 to M.J.P.); and the University of Utah Consortium for Families & Health Research Fund award to A.B. This material is the result of work supported with resources and the use of facilities at the Salt Lake City VA medical center in Salt Lake City, UT.

## Conflicts of interest

None declared.

## Data Availability

The data that support the findings of this study are available on request from the corresponding author, upon approval of the required data use agreement. The data are not publicly available due to privacy or ethical restrictions.
